# Recent Technological Advances in Using Mouse Models to Study Ovarian Cancer

**DOI:** 10.3389/fonc.2014.00026

**Published:** 2014-02-13

**Authors:** Carrie Danielle House, Lidia Hernandez, Christina Messineo Annunziata

**Affiliations:** ^1^Women’s Malignancies Branch, National Cancer Institute, Bethesda, MD, USA

**Keywords:** mouse models, serous epithelial ovarian cancer, imaging techniques, immune therapy, cancer stem cells, reporter, metabolite profiling

## Abstract

Serous epithelial ovarian cancer (SEOC) is the most lethal gynecological cancer in the United States with disease recurrence being the major cause of morbidity and mortality. Despite recent advances in our understanding of the molecular mechanisms responsible for the development of SEOC, the survival rate for women with this disease has remained relatively unchanged in the last two decades. Preclinical mouse models of ovarian cancer, including xenograft, syngeneic, and genetically engineered mice, have been developed to provide a mechanism for studying the development and progression of SEOC. Such models strive to increase our understanding of the etiology and dissemination of ovarian cancer in order to overcome barriers to early detection and resistance to standard chemotherapy. Although there is not a single model that is most suitable for studying ovarian cancer, improvements have led to current models that more closely mimic human disease in their genotype and phenotype. Other advances in the field, such as live animal imaging techniques, allow effective monitoring of the microenvironment and therapeutic efficacy. New and improved preclinical mouse models, combined with technological advances to study such models, will undoubtedly render success of future human clinical trials for patients with SEOC.

## Introduction

Mouse models provide a wealth of information for understanding tumor biology. Through the validation of *in vitro* findings, cancer progression, regression, and relapse in the physiological setting is better understood. The continued high mortality associated with serous epithelial ovarian cancer (SEOC) underscores a need for tailored disease models and improved technology to study such models. Several recent advancements promise to facilitate the success of preclinical models in refining our understanding and treatment of SEOC. This mini-review will focus on the latest mouse models of ovarian cancer and improved technologies for using these models to study SEOC initiation, progression, relapse, and therapeutic response.

Ovarian cancer is the most common cause of gynecological mortality in the United States, accounting for more than 14,000 deaths annually (1). Most patients initially respond favorably to platinum-based therapy, however, there is a high incidence of recurrent, chemoresistant disease. Our knowledge of the clinical and molecular attributes of epithelial ovarian cancer has improved greatly over the last few decades, but survival rates for women with this disease remain low. This is partially explained by the inability of clinical trials to replicate the therapeutic response observed in animal models. So far, about one-third of highly cited animal studies saw success in human trials, however, improvements in study design and data interpretation should increase that figure (2, 3). Animal models in the personalized medicine era highlight the availability of affordable genomic sequencing and molecular profiling. As the pharmaceutical industry relies heavily on mouse models, such new refinements will be critical for producing reliable preclinical data on personalized ovarian cancer therapeutic approaches.

In order to generate accurate models, the biology of the disease must be understood. High grade SEOC is thought to arise in a rapid fashion *de novo* from the surface epithelium of the ovary or from the mucosa of the fallopian tube (4, 5). The remaining ovarian carcinomas, categorized as low grade, follow a stepwise adenoma-carcinoma sequence (4, 6). Whether high or low grade, SEOC usually does not reach clinical detection until late stage where it has spread well beyond the ovaries. This feature has hampered efforts to identify the site of origin and understand the pathophysiology of SEOC. Most existing mouse models of SEOC present a disseminated abdominal phenotype, which closely resembles late metastatic disease, and therefore may only provide a good model for therapeutic response in the “average” patient. Some recent mouse models provide a phenotype of early progressive disease coming from defined genetic abnormalities identified from patient subtypes.

## Mouse Models of EOC

An extensive analysis of every mouse model is beyond the scope of this mini-review, however, a summary of recent advances in mouse models of ovarian cancer to place the technological advances in context is presented here. Several recent reviews are available detailing epithelial ovarian cancer experimental models (7–9). Mouse models of ovarian cancer generally fall into one of three categories (xenograft, syngeneic, genetically engineered), the most suitable being dependent on the information being sought.

### Xenograft models

A human tumor xenograft is the most widely used mouse model in which human tumor cells are transplanted under the skin (subcutaneous), into the abdominal cavity (intraperitoneal), or into the organ of origin (orthotopic) of an immune-compromised host. While intraperitoneal and orthotopic injections can mimic metastatic dissemination, subcutaneously injected cells are largely limited to tumor formation at the site of injection. The unique presence of a bursa, a sac encapsulating the ovaries and fallopian tubes, allows for intra-bursal injections in mice (10, 11). This technique permits the study of early, localized disease, tumor cell invasion, and dissemination in a more biologically relevant order of events (12).

Xenograft models are particularly useful for evaluating tumorigenesis in a timely fashion (13, 14). Within a few weeks, tumor formation can be measured *in vivo* with histology representative of the tumor of origin (12, 15, 16). Importantly, the pattern of spread to the ascites, liver, and spleen, typical in human disease, is replicated in many of these models and depending on the cell line used, tumors representing the different histological subtypes of epithelial ovarian cancer can be produced (8, 12). Xenografts are versatile and often used in parallel with *in vitro* studies to generate a majority of preclinical data.

Although quite valuable, xenografts carry important limitations. One major disadvantage is the lack of immune response inherent in these models. Nude mice are athymic and therefore have a limited T cell response, and severe combined immunodeficiency (SCID) strains lack both T and B cell responses. Because tumors can promote anti-tumor responses such as lymphocyte and macrophage infiltration, these models may not accurately represent disease progression and therapeutic response observed in otherwise immune-competent individuals (17–19). Furthermore, these models are not suitable for studying immunotherapy or mechanisms involving host–cell interactions. Cell line-derived xenografts have had little success in predicting therapeutic response in patients, thereby emphasizing a need for improvements to current models.

An alternative to traditional cell line-derived xenograft models involves the direct transfer of tumor fragments from individual patients. Minced fragments are delivered via orthotopic or intraperitoneal injection into immune-deficient mice to create “xenopatients” or tumor grafts (8, 20). Successful engraftment is higher in SCID mice compared to nude mice, likely due to the suppression of both cellular and humoral immunity (20–23). Several reports have demonstrated that tumor grafts stably maintain the histopathology, immunophenotype, and heterogeneity of the original tumor through multiple passages (21–26). Moreover, these models have the capacity to recapitulate the same therapeutic properties observed in patients (20, 25, 26). The better predictive response value makes these models superior to traditional cell line-based xenografts generated using a suspension of mostly homogenous cells. Engraftment of the native stromal extracellular matrix that would normally accompany a tumor graft may provide the most suitable microenvironment for replicating the biology of the original tumor. This feature renders tumor grafts more suitable for studying early metastasis, as it relies on dissemination of cells from a tumor fragment rather than dispersion of cells from a suspension (8). Thus, patient-derived tumor grafts provide a means to model inter-patient heterogeneity known to exist across high grade SEOC, and to study tumor evolution through exposure to therapy. Tumor grafts, although promising, are not without their own challenges. Generating a mouse model using a tumor graft is labor intensive and expensive and, as in traditional xenografts, the mice are immunocompromised; consequently immune responses cannot be studied. Although well suited for clinicians and personalized medicine, access to patient tumor samples can be challenging for many basic and translational investigators. Some research teams have generated banks of tumor grafts to make these models more accessible (20, 23, 24).

### Syngeneic models

Some challenges of xenograft models can be overcome using syngeneic mouse models, wherein tumors are established in immune-competent mice using cells from the same strain. In one of the most widely used syngeneic models, generated by Roby et al., ovarian surface epithelial cells isolated from immune-competent mice were repeatedly passaged *in vitro* until transformation occurred, and subsequently injected back into the same strain (27). Other syngeneic models have been created using genetically modified cells (28, 29) and highly metastatic cell lines stably expressing luciferase for monitoring disease (30). The histopathologic characteristics observed in the tumors of these models including the presence of papillary structures, nuclear atypia, and malignant ascites, closely resemble those seen in humans (29, 31).

The major advantage of this model is that the mice have an intact immune system; therefore the anti-tumor immune response can be examined and the risk of infection is minimized (19, 32). Syngeneic models provide the opportunity to study the tumor microenvironment, epithelial–stromal cell interactions, tumor-secreting factors, immune cell infiltration, and vasculature (28, 29, 31, 33). This model, however, is completely derived from the animal system and therefore may not mirror every element of human cancer. Although human and mouse tumors share similar features, the complexity of human disease coupled with the heterogeneity of cancer make it difficult to translate findings (34).

### Genetically engineered models

Genetically engineered mouse models (GEMMs) are immune-competent mice with genetic defects introduced using RNA interference, inducible gene expression, viruses, or DNA recombination techniques. GEMMs provide a means for investigating the role of genetic alterations in cancer development. These models allow researchers to control and direct gene expression, which can be limited to the tissue of interest using a tissue-specific promoter to introduce the desired genetic alteration, or expressed throughout the organism using germ-line mutations (35). Furthermore, regulation of gene expression in the presence or absence of tetracycline and its receptor allows for inducible gene expression systems and provides the flexibility to turn on or off gene(s). For example, transgenic mice carrying both the tetracycline-regulated transcriptional transactivator and its respective binding site linked to a gene of interest permits amplified expression of that gene. If mice are provided with the tetracycline antibiotic in their drinking water, this expression is reversibly suppressed. Thus, GEMMs provide opportunities to identify which genes are necessary for disease progression, regression, and/or resistance to treatment.

Extensive analyses of human ovarian cancer specimens have identified several genetic alterations associated with malignancy including *TP53, C-MYC, K-RAS, AKT*, and *BRCA1* and *BRCA2* (36–38). Subsequently, several genetically modified ovarian cancer models, summarized in Table 1, have been developed to explore the contribution of these different aberrations to ovarian cancer development (39–44).

**Table 1:** **GEMMs for ovarian cancer**.

Original reference	Genes altered	Ovarian specific expression	Cancer histology	Comments
(39)	*p53, c-Myc, Kras, Akt*	Oncogenes were delivered *in vitro* into ovarian epithelial cells from a transgenic *p53*-deficient mouse; modified cells were then introduced into ovarian bursa of the same mouse	Ovarian carcinoma	Illustrates necessity for p53 deficiency in combination with at least two other oncogenes for tumor induction
(41)	*p53, Rb1*	Adeno-Cre was introduced into ovarian bursa of transgenic mice carrying floxed alleles	EOC	p53 and Rb1 cooperate in EOC development
(40)	*p53, Brca1, c-Myc*	c-Myc and Cre were retrovirally delivered into ovarian explants from floxed *Brca1* and *p53* transgenic mice; modified cells were then introduced i.p. into recipient syngeneic mice	SEOC	Identifies the requirement for *Myc* in p53 and Brca1-induced transformation
(42)	*Pten, Apc*	Adeno-Cre was introduced into ovarian bursa of transgenic mice carrying floxed alleles	OEA	Illustrates the role of Wnt and PI3K signaling in development of ovarian endometrioid adenocarcinoma (OEA)
(43)	*Pten, Kras*	Anti-Mullerian hormone receptor directed Cre-expressing mice crossed with mice carrying floxed alleles	Low-grade serous adenocarcinoma	Demonstrates role of Kras transformation and loss of Pten for elevated p53 levels and associated low-grade phenotype
(44)	*p53, Rb, Brca1 or Brca2*	Adeno-Cre was introduced into ovarian bursa of transgenic mice carrying floxed *p53* and *Brca* alleles and *Rb* deficiency directed to epithelium by Keratin18 promoter for T-antigen expression	SEOC	Genetic modifications recapitulate human SEOC stages

Although GEMMs are labor-, time-, and resource-intensive, they provide information that cannot be attained in xenograft or syngeneic models. Early tumorigenesis and genetic events leading to tumor initiation, maintenance, and relapse can be analyzed. The flexibility provided by genetic manipulation permits the study of different mutation combinations. These models are ideal for target validation, treatment response, and chemoprevention (45). The major challenge with this model is the scarcity of tissue-specific promoters in ovarian surface epithelium or distal fallopian tube. It is also challenging to accurately replicate the contribution of genetic elements given that genes over-expressed in mice are often at non-physiological levels or deleted throughout the organism (46). GEMMs may fail to recapitulate the genetic complexity of human SEOC, and the varied genetic background of different mouse strains can influence findings and conclusions (8).

## Technological Advances in Using Animal Models

### Reporters

Most ovarian cancer cell lines can be stably transfected with a fluorescent and/or bioluminescent reporter for monitoring tumor cell growth and dissemination, pathway activity, and receptor interactions.

This technology has been adapted to xenograft and syngeneic mouse models of ovarian cancer. For example, NF-κB activity was tracked in a syngeneic model of SEOC to confirm that activation correlated with progression and influenced immune cells of the microenvironment (47). Similarly, reporter-tagged tumor cells can be used to monitor tumor response in real-time using digital imaging following systemic targeted therapy (48–50). Using reporters in live animals to track tumor cell dissemination allows for studying cancer progression and therapeutic response, especially in syngeneic models where the immune response is integral.

Luciferase complementation-based assays measure receptor activation and protein interactions using monomeric enzyme components that have enzymatic activity only when complementation is induced by the interaction of binding partners or small molecules (51). Activation is proportional to the production of light that occurs upon complementation. The flexibility of this technology allows detailed quantitative measurements of complexes, assessment of nuclear translocation, and identification of pathway modulators (52). For example, this assay was successfully implemented for live imaging of the chemokine, CXCL12, interacting with its receptor, CXCR4, in animal models of ovarian cancer (53, 54).

### Imaging

Quantitative measurements of late-stage disease in ovarian cancer models are challenging due to the presence of varying levels of ascites and the poor correlation between total body weight gain and tumor burden. Diagnostic imaging is a reproducible means to quantify tumor mass, monitor tumor progression, and interrogate the tumor microenvironment. Imaging techniques used in the clinic [e.g., magnetic resonance imaging (MRI), computed tomography (CT), positron emission tomography (PET), ultrasound] have been adapted for use in animals (55, 56). These modalities are especially informative as they can be performed in intact living animals. Interval imaging reduces the number of animals needed for experiments as measurements are taken without sacrificing the animal. The major challenge to imaging ovarian cancer in animal models, as in humans, is the difficulty in detecting early disease; by the time mice begin to show signs of morbidity the cancer has often spread beyond the ovaries and throughout the peritoneum.

Positron emission tomography imaging is a standard diagnostic radiological technique commonly used to monitor drug action in cancer patients. This modality allows the measurement of metabolic activity in cancer cells and is especially useful in quantitative monitoring of tumor response to anti-cancer therapies (56). PET imaging can assess targeted therapies in both transgenic (57) and xenograft (58) models of ovarian cancer.

Ultrasound imaging is another common tool used in small animal models and is often combined with other imaging techniques for a more comprehensive analysis (57). Ultrasound is cost-effective and convenient for measuring individual tumors in live animals (59). Doppler ultrasonography can measure changes in blood flow and angiogenesis associated with disease progression or response to anti-angiogenic therapy (59, 60).

Magnetic resonance imaging with gadolinium-based contrast agent permits high-resolution serial imaging with minimum scanning duration, allowing quantification of tumor volume over time. MRI data are comparable to caliper-based measurements taken at necropsy. This longitudinal imaging protocol is well suited for monitoring therapeutic response (61). MRI can also be combined with fluorescence molecular tomography (FMT) to monitor tumor-specific biology, such as protease and integrin activity (62). When coupled with a reporter gene such as ferritin heavy chain (FHC), MRI can evaluate recruitment of other cell types, such as fibroblasts, to the tumor site (63). Alternatively, MRI combined with magnetic resonance spectroscopy (MRS) can characterize tumor physiology and metabolic profiles over time (64).

### Metabolic profiling

Measurement of metabolites and their intermediates can illustrate the response of an organism to a genetic manipulation or therapy. Metabolites are small, low molecular weight analytes and include amino acids, oligopeptides, sugars, fatty acids, and various intermediates of biochemical pathways, in contrast to large proteins and nucleotides that are assessed using proteomics and genomics, respectively (65). Nuclear magnetic resonance (NMR) spectroscopy, liquid and gas chromatography, and mass spectrometry (MS) are generally used to analyze serum, urine, or tissue extracts. Such measurements provide insight into drug mechanisms and toxicities. Metabolic profiles represent a snapshot of the biochemical reactions occurring at a point functionally downstream of genome, transcriptome, and proteome (65).

Commonly used in human studies, (66, 67) this technology was adopted in a GEMM of SEOC. The metabolic profile overlapped with human SEOC and showed a temporal correlation with disease progression (44, 68), highlighting the feasibility of metabolic profiling for identifying biomarkers and monitoring treatment response in animal models (44).

### Tumor-initiating cells

The cancer stem cell (CSC) or tumor-initiating cell (TIC) hypothesis suggests that a small population of chemoresistant cells reside in the tumor, capable of reconstituting the tumor. These cells share properties of normal stem cells, such as self-renewal and multi-potency. Given the high recurrence of ovarian cancer, the TIC hypothesis is an attractive model for explaining ovarian cancer relapse.

Mouse models have been especially useful in evaluating TICs. When injected into mice, these cells must recapitulate the heterogeneity of the original tumor. Animal models are essential for defining TICs and for evaluating drugs and pathways important for eradicating these cells. Patient-derived xenografts might allow further characterization of the frequency of TICs in human tumors, and their relevant biomarkers.

A number of markers have been used to identify and isolate ovarian cancer TICs including CD133, CD44, CD117, and ALDH activity; however it is unlikely that a single marker defines ovarian TICs (69, 70). Several studies have demonstrated heterogeneous tumor formation in xenograft mice after subcutaneous injection of sorted ovarian cancer cells from primary tumors, cell lines, or ascites (71–74). TICs have also been propagated *in vitro* using low attachment culture plates and specialized serum-free media to enhance the formation of multicellular spheroids with stem-like features (69, 73).

Although much research has focused on characterizing tumorigenesis of human TICs in xenograft models, recent studies evaluated endogenous TICs in mice (75–77). Syngeneic or GEMMs offer the possibility of studying the role of the immune system in TIC biology. Furthermore, with direct or indirect labeling of the TICs, each of these models can facilitate tracking of the cells to monitor tumor initiation and dissemination.

### Immune therapies

The role of the immune system in ovarian cancer is studied extensively using animal models (19). Representing a robust predictor of outcome, tumor-infiltrating lymphocytes are associated with better survival for ovarian cancer patients (78, 79). Immune therapies involving vaccines, dendritic cell therapy, engineered T cells, and immune modulators thus hold promise for ovarian cancer treatment (80–86).

Current goals aim to enhance the anti-tumor immune response through increased immune activation and decreased immune suppression. Programed death-1 (PD-1) and CTL antigen-4 (CTLA-4) signals silence the immune response in tumors. A syngeneic mouse model of ovarian cancer showed that simultaneously blocking these pathways enhanced T cell infiltration into the tumor and increased long-term survival (81). A related model found that the therapeutic effect of gemcitabine is limited because of the immunosuppressive network of CTLA-4 (83). Gemcitabine plus anti-CTLA-4 antibody exhibited synergy in a strong anti-tumor immune response. Likewise, anti PD-1 therapy shows synergism with a variety of immunotherapies or vaccines (86). These findings have translated well and are currently under evaluation in the clinic.

Genetically modified T cells engineered to over-express receptors for tumor-associated antigens have shown great success in mouse models of ovarian cancer (87, 88). This emerging technology is a logical avenue for ovarian cancer, an apparently immunogenic disease where T cell infiltration is associated with improved survival (19, 88).

## Conclusion

**Figure 1:**
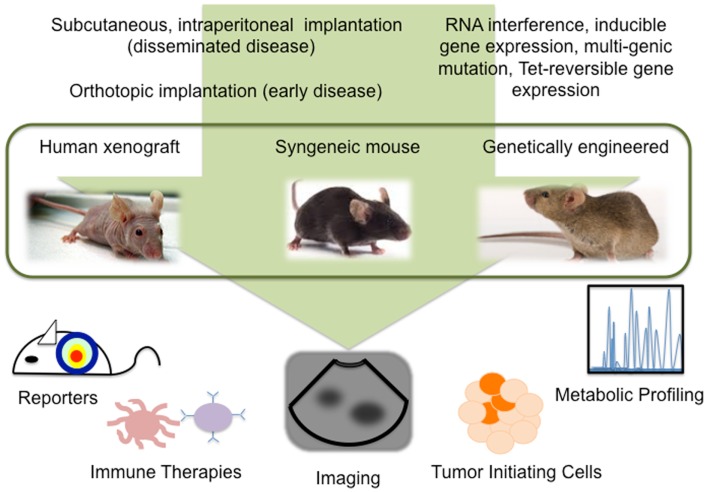
**Technological advances in mouse models allow detailed study of ovarian cancer biology**.

Despite our progress in understanding ovarian cancer biology, there remains a high mortality associated with this disease. Exciting advances in reporter assays, live imaging, metabolomics, TICs, and immune therapies, provide new information about the tumor microenvironment and further our understanding of SEOC development, progression, and recurrence (Figure 1). Further refinement of mouse models of ovarian cancer, an awareness of the limitations each model presents, and taking advantage of the technologies available to study these models will undoubtedly expedite the success of new treatments.

## Conflict of Interest Statement

The authors declare that the research was conducted in the absence of any commercial or financial relationships that could be construed as a potential conflict of interest.
